# Acute Neuropixels Recordings in the Marmoset Monkey

**DOI:** 10.1523/ENEURO.0544-23.2024

**Published:** 2024-05-14

**Authors:** Nicholas M. Dotson, Zachary W. Davis, Patrick Jendritza, John H. Reynolds

**Affiliations:** ^1^The Salk Institute for Biological Studies, La Jolla, California 92037; ^2^Department of Ophthalmology and Visual Sciences, John Moran Eye Center, University of Utah, Salt Lake City, Utah 84132

**Keywords:** acute recording, electrophysiology, laminar, marmoset, Neuropixels, single unit

## Abstract

High-density linear probes, such as Neuropixels, provide an unprecedented opportunity to understand how neural populations within specific laminar compartments contribute to behavior. Marmoset monkeys, unlike macaque monkeys, have a lissencephalic (smooth) cortex that enables recording perpendicular to the cortical surface, thus making them an ideal animal model for studying laminar computations. Here we present a method for acute Neuropixels recordings in the common marmoset (*Callithrix jacchus*). The approach replaces the native dura with an artificial silicon-based dura that grants visual access to the cortical surface, which is helpful in avoiding blood vessels, ensures perpendicular penetrations, and could be used in conjunction with optical imaging or optogenetic techniques. The chamber housing the artificial dura is simple to maintain with minimal risk of infection and could be combined with semichronic microdrives and wireless recording hardware. This technique enables repeated acute penetrations over a period of several months. With occasional removal of tissue growth on the pial surface, recordings can be performed for a year or more. The approach is fully compatible with Neuropixels probes, enabling the recording of hundreds of single neurons distributed throughout the cortical column.

## Graphical abstract

**Figure:**
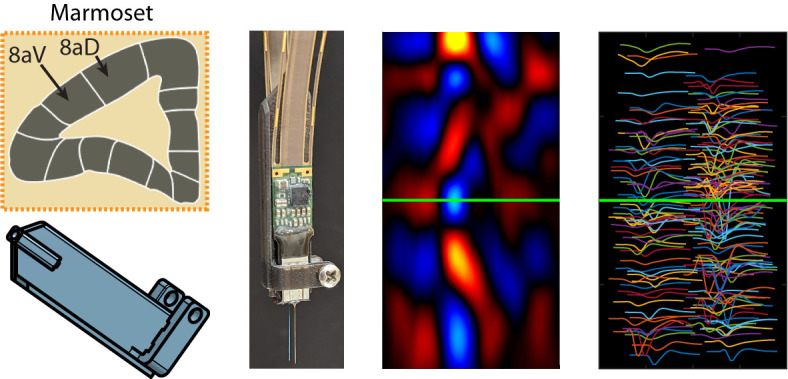
Visual Overview

## Significance Statement

The cerebral cortex of the macaque monkey is extensively folded, which poses a major problem for studying laminar computations in many cortical areas. Marmosets, however, have a smooth brain that allows for simultaneous recordings from all layers of the cortex in areas that are buried deep in sulci in the macaque. In this manuscript, we describe an artificial dura system that utilizes the state of the art in high-density probes, Neuropixels. This system enables us to easily insert multiple Neuropixels into the marmoset cortex normal to the cortical surface permitting repeated laminar recordings for up to a year or more.

## Introduction

**Figure 1.**
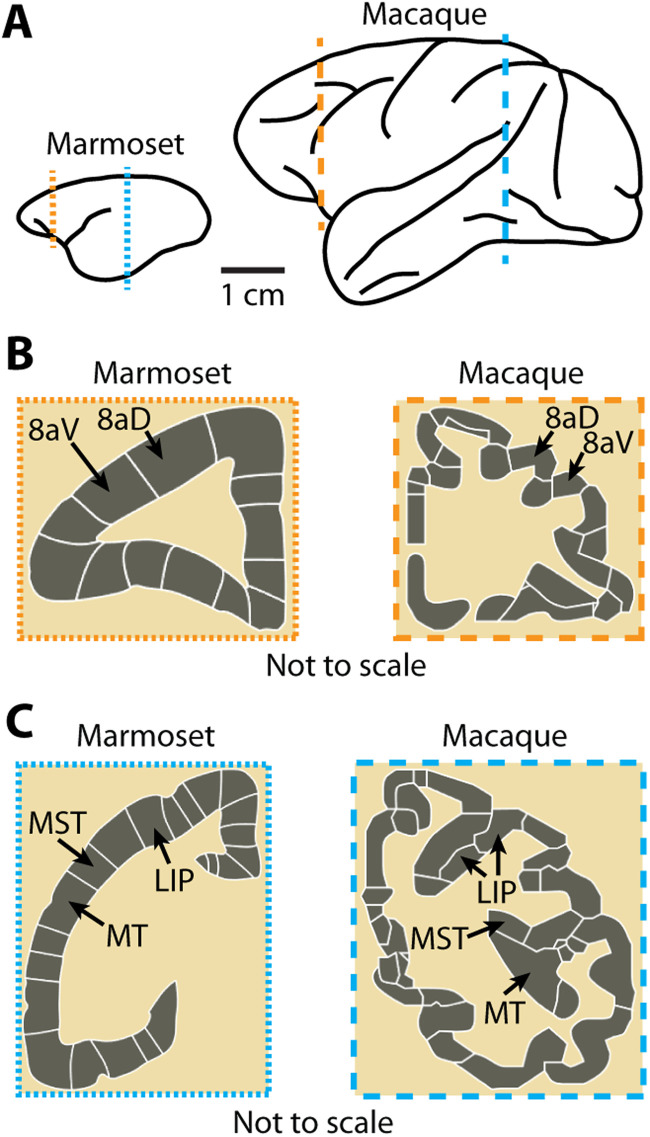
The near absence of sulci in the marmoset makes the cortex highly suitable for laminar recordings. ***A***, Trace drawings of the marmoset and macaque monkey brains illustrating their relative sizes and the differences in the prevalence of sulci. The marmoset cortex is mostly smooth, while the macaque cortex is highly convoluted making it difficult to record perpendicular to the surface in many locations of the cortex. ***B***, ***C***, Digital reconstructions (not to scale relative to each other) of coronal slices further illustrate the relative ease of making laminar recordings in the marmoset compared with the difficulties with performing laminar recordings in the macaque monkey. Cortical areas 8aV, 8aD, MT, MST, and LIP are highlighted to show the differences in position created by sulci (images in ***B*** and ***C*** are from the scalablebrainatlas.incf.org; Paxinos et al., 2000; Van Essen, 2002; Tokuno et al., 2009; Paxinos et al., 2012; Bakker et al., 2015).

Recent advancements in electrophysiological recording tools have made it possible to study cortical function at unprecedented scales, including simultaneous recordings of hundreds to thousands of neurons across the depth of cortical layers using Neuropixels probes (Steinmetz et al., 2018; Siegle et al., 2021). In the macaque monkey, many of these tools have limited utility for studying laminar processing because of the extensive gyrification of the neocortex, making it challenging in many brain areas to reliably obtain perpendicular penetrations of the cortex with linear electrode arrays (Fig. 1). The marmoset has been gaining interest as a model organism for studying cortical function due to its lissencephalic (smooth) cortex and its ability to perform complex visual tasks (Mitchell et al., 2014; Solomon and Rosa, 2014; Selvanayagam et al., 2019; Cloherty et al., 2020; Davis et al., 2020, 2023; Feizpour et al., 2021; Jendritza et al., 2021, 2023; Dotson et al., 2023; Kell et al., 2023; Piza et al., 2023). However, there are still numerous technical considerations that can pose challenges to maximizing the utility of the marmoset cortex for neuroscience research. The marmoset skull, compared with that of a macaque, is small and thin (∼1 mm thick) and therefore requires the use of lightweight materials and small parts. Similar to the macaque monkey, the marmoset dura is opaque and difficult to penetrate with laminar probes, often requiring its removal. However, removing the dura in order to access the brain introduces risks of infection or damage and results in the formation of coarse granulation tissue that must be periodically removed (Sadakane et al., 2015).

Here we describe a technique for repeated acute recordings of laminar cortical activity using one or two Neuropixels probes (short, linear, and sharpened nonhuman primate version) in the marmoset neocortex. Our method utilizes an artificial dura (AD) system similar to the ones previously used in macaque monkeys (Shtoyerman et al., 2000; Arieli et al., 2002; Chen et al., 2002; Gray et al., 2007; Ruiz et al., 2013; Nassi et al., 2015; Nandy et al., 2017; Franken and Reynolds, 2021) but modified to suit the specific challenges that arise due to the smaller size of the marmoset. The recording chamber provides mounts for attaching a microdrive and houses a removable insert that is fitted with an AD. The AD is made from transparent silicone elastomer, providing optical clarity of the cortical surface, thus permitting the insertion of Neuropixels and other high-density linear electrode arrays perpendicular to the cortical surface while avoiding blood vessels. The chamber is sealed using a silicone gasket which greatly reduces the risk of infection and can be flexibly designed to accommodate a variety of experimental aims, including other recording and stimulation techniques, such as optical imaging, optogenetics, and semichronic microdrives.

## Materials and Methods

### Part designs

Any available part designs or further details can be provided upon request.

### Animals

The AD system was implanted on four male marmosets.

### Chamber implant

All chambers were implanted in a similar manner. For the prefrontal cortex (PFC) implant, the chamber is placed over the PFC using the midline and the orbital arch as landmarks. First, an incision is made down the midline of the skull and the underlying tissue is removed from the bone. A thin layer of dental bonding agent (OptiBond Universal, Kerr) is then applied to the skull. Next, a craniotomy is made and several screws are placed around the perimeter. The chamber is then placed over the craniotomy and fixed in place using a fast-curing acrylic (Jet Acrylic, Lang Dental). Following the durotomy, the insert with the attached AD is lowered into the chamber. The durotomy does not necessarily have to be performed during the same surgery. For one of the PFC implants, the durotomy was performed several months after the chamber implant. With the occasional surgical removal of granulation tissue growth, acute Neuropixels recordings can be made for over a year.

### Neural recordings

Prior to neural recordings, marmosets are trained to freely enter custom-made marmoset chairs, which are then positioned in front of a calibrated and gamma corrected LCD monitor (ASUS VG248QE; 100 Hz refresh rate; 75 cd/m^2^ background luminance). Eye position is measured with a video-based eye tracker (ISCAN ELT-200, 500 Hz sampling rate). Stimulus presentation and behavioral control are performed using MonkeyLogic (Asaad and Eskandar, 2008; Hwang et al., 2019). Digital and analog signals are coordinated through National Instrument DAQ cards (NI PCI6621) and BNC breakout boxes (NI BNC2090A). Neuropixels recordings are acquired using the PXIe Acquisition Module (Neuropixels), and SpikeGLX acquisition software. We used either the chamber or a stainless steel bone screw placed in the skull as the ground and the built-in tip reference on the Neuropixels probe as the reference signal. A synchronization pulse is sent to both the PXIe Acquisition Module and the National Instrument DAQ card. Spike sorting is performed using Kilosort 2.0 and Phy (Pachitariu et al., 2016; https://github.com/cortex-lab/phy).

The chamber is rinsed with sterile saline before and after each recording. Before insertion, the probes were disinfected by placing them in a UV-clave (25 min) and dipping them in isopropyl alcohol. We monitored the probes during the insertion process using a miniature digital microscope. Probes were first lowered down to the AD using the coarse manipulator on the microdrive. Typically, the AD would dimple slightly and then the probes would pass through. On no occasion did the AD stop the probes. Typically there was a small gap between the AD and the pial surface. So, the probe had to be lowered further before hitting the pia. The process of penetrating the pia varied depending on the time since the last pial surface cleaning. Shortly after removing the dura or after cleaning the pia, the probe would smoothly enter the brain (∼1 month), with no visual evidence of distortion. After 2–3 months of tissue growth on the pial surface, the probes would often bend slightly during penetration. Pial growth would also cause the brain to dimple when inserting the probe. Eventually, it would become difficult or impossible to lower the probes into the brain, in which case recordings would cease until after pial growth was removed.

Once neural activity was observed on the channels near the tip of the probe, it could be verified that the probe had entered the brain. After the probes entered the brain, they were lowered in increments of ∼250 microns at a rate of 2.5–5 microns/s, with a 1–5 min wait period. This process would be repeated until the probe was lowered ∼2,500 microns beyond the depth at which neural activity was first observed. Probes were then retracted 200–300 microns. This helped to relieve any dimpling caused during probe insertion. Throughout this process, progress was monitored by observing the probe through the microscope and by watching neural signals. Often, neural activity would march up the probe as it was lowered. After lowering the probe and allowing it to settle for 30 min, recordings are then conducted for ∼2 h. Probes were withdrawn from the brain at a slightly faster speed (∼25 microns/s). Immediately after withdrawal, the probes were soaked in a 1% Tergazyme solution for ∼2–18 h. Next, they were soaked in DI water for 15–30 min and then prepared for another recording or stored in a closed container.

Viable recording periods were typically ∼3 months. We often several months after tissue growth prevented electrodes from penetrating before removing tissue growth. This delay makes the tissue overlying the pial surface less friable and easier to remove. For example, in one animal, tissue growth was removed 6 months after the durotomy and then again after another 9 months. Tissue growth would occur over time, and given long enough bone would begin to form. After 3–4 months, a bone shelf would form around the edge of the craniotomy, which can eventually seal the entire craniotomy. This growth, however, is easily removed using forceps or with careful use of a surgical drill. A chamber that goes further into the bone or a longer insert may help prevent this process. With the technique described here, the AD should not need to be replaced. If it does need to be replaced, this is typically due to the AD coming detached from the rim of the insert, which can occur after repeated penetrations. However, generally, if the AD is replaced at all, it is not more than once during a recording period. In some instances, we would use a short insert after surgery to accommodate any swelling that may occur and then later replace it with a longer insert. When the insert or AD needs to be replaced, we rinse multiple times with sterile saline inside the chamber before replacing the AD. We have not observed any infections above or below the AD.

### Laminar identification and reconstruction methods

Current source density (CSD) plots are calculated by taking the second spatial derivative of the average stimulus-evoked local field potential (LFP; Mitzdorf and Singer, 1978; Mitzdorf, 1985; Franken and Reynolds, 2021; Davis et al., 2023). Full-screen, full-luminance flashes (20 ms) were presented when the animal fixated within a 5 dva window at the center of the screen. A 5 s intertrial interval followed each flash. Approximately 50–100 trials were recorded for each session. Due to the close spacing of contacts on the Neuropixels probes, the signals were spatially averaged over many channels using a 2D Gaussian kernel (120 channels, 20 ms, with SD = 12). An alternative technique of taking the average of chunks of 10 channels provided a nearly identical result. In this case the signals were spatially averaged using a smaller 2D Gaussian kernel (three channels, 5 ms, with SD = 1.1). These CSDs are similar to the ones generated using probes with 100 µm spacing between contacts. Plots are smoothed using bicubic (2D) interpolation before plotting (MATLAB function interp2 with option cubic). CSD data are presented in arbitrary units because our primary objective was only to identify the input layer using the relative timing and location of sources and sinks and therefore did not require properly calculating the CSD values using the conductance of the cortex.

To calculate the spike–field relationships, we used 15 min of data starting at the beginning of the session. We used the mean LFP and all the spikes from groups of 10 channels (channels 1–10, 11–20, and so on). The LFP (1 kHz sampling rate) was bandpass filtered 5–35 Hz (four-pole Butterworth filter, zero-phase), and then the Hilbert transform was used to estimate the instantaneous phase angle at each time point. For each pair of signals (LFP and spike), the phase angle of the LFP at the time of each spike was binned. The mean phase angle of the spike–phase distribution was calculated using the circular mean function (circ_mean.m) in the Circular Statistics Toolbox for Matlab (Berens, 2009). Notably, for most Neuropixels recordings, we attached the ground wire to the chamber or a stainless steel bone screw and used the built-in tip reference on the probe as the reference channel. Due to this referencing scheme, we found it necessary to re-reference to the common average (i.e., subtract the average LFP signal) in order to recover the common spike–field relationships observed across cortical areas and species (Davis et al., 2023).

## Results

### AD recording system for the marmoset

**Figure 2.**
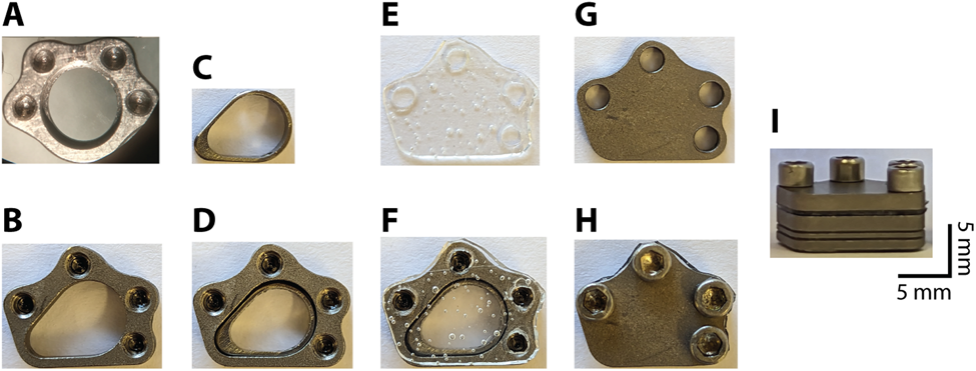
Recording chamber components. ***A***, Image of circular recording chamber used for recordings (top view). ***B***, Image of egg-shaped recording chamber designed for PFC recordings (top view). ***C***, Insert for PFC chamber (AD not attached). ***D***, Chamber with the insert inside. ***E***, Image of the silicone gasket used to seal the chamber. ***F***, Image of the gasket on top of the chamber. ***G***, Image of the cap for the PFC chamber. ***H***, Image of the chamber with gasket, cap, and four screws fixing the cap in place (top view). ***I***, Side view of the PFC chamber with gasket and cap. The scale bars in ***I*** apply to all images.

The AD system consists of a chamber, an insert with the AD adhered to the bottom, and a cap with a silicone gasket (Fig. 2). The chambers are machined out of titanium, and multiple designs have been implemented to record either in area MT (Fig. 2*A*) or the PFC (Fig. 2*B*). The maximum inner dimension of the chamber, not including the insert, ranges from 6 to 9.5 mm depending on the chamber design, providing access to multiple cortical areas. The total height, including the chamber (3.1 mm), housing cap (1.6 mm), and gasket (0.5 mm) is 5.2 mm. The housing cap is secured with four screws placed strategically around the chamber perimeter. A thin skirt that extends beyond the bottom of the chamber passes into the craniotomy to ensure a tight fit. The total weight of the chamber, insert, gasket, cap, and screws is ∼2.4 g.

The shape of the chamber is specifically customized to fit the constraints of the implantation site. For example, the circular chamber designed for MT (Fig. 2*A*) has the screw holes placed more dorsally to reduce the impact on the ventral aspect of the skull near the marmoset ears. The egg-shaped chamber (Fig. 2*B*) was shaped to provide access to the more anterior PFC regions while reducing the implant's footprint near the orbitofrontal bone and eyes. The chambers are also versatile and can be placed over other brain areas, with the chamber choice largely depending on the shape of the skull above the areas of interest. For example, the circular chamber is more suitable for recording from cortical areas below portions of the skull with more curvature such as ventral PFC targets.

**Figure 3.**
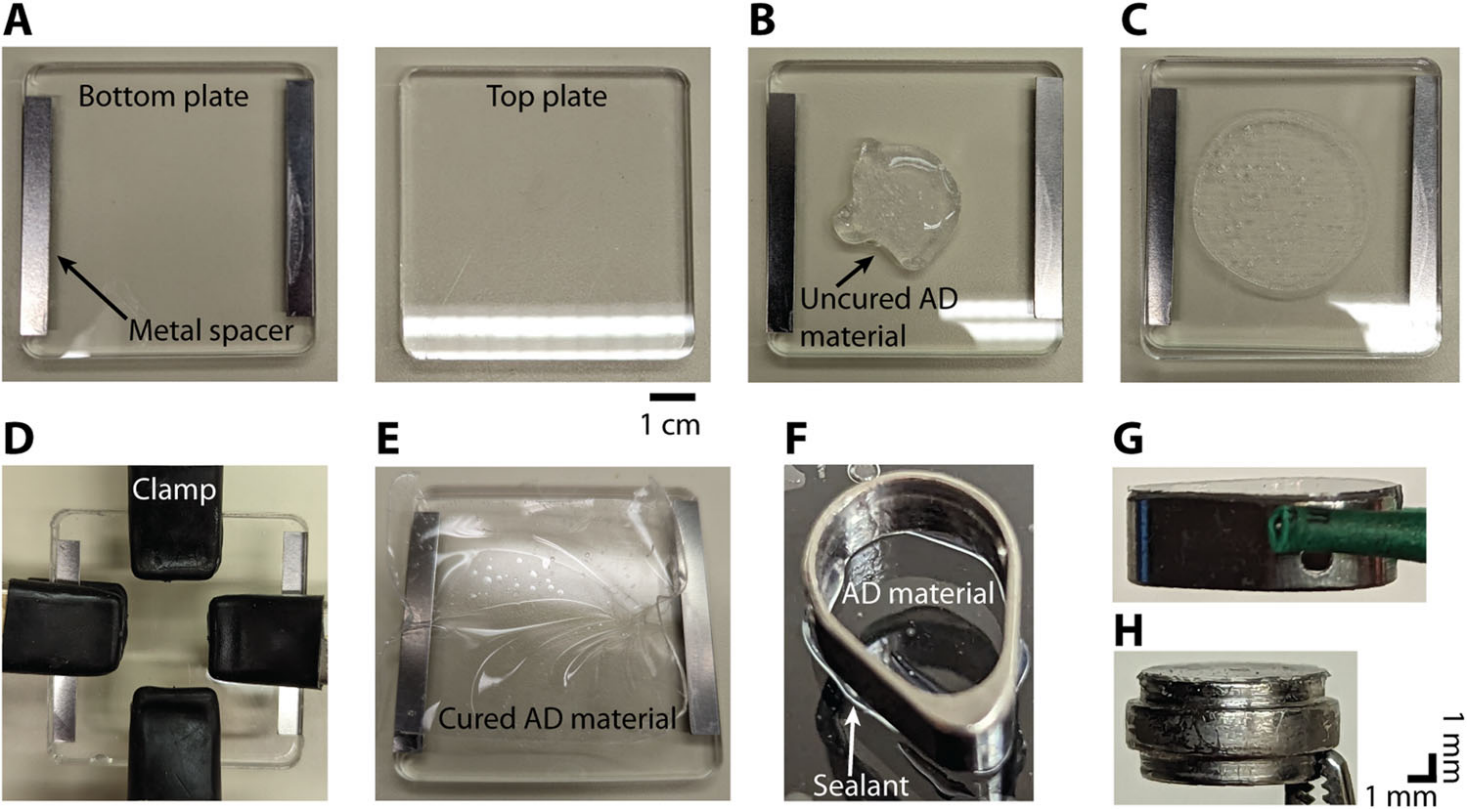
Procedure for making the AD material and adhering it to the insert. ***A***, Two acrylic plates are used to cure the AD material into a thin sheet. Metal spacers are attached to the bottom plate (left) in order to create a gap of 400 μm between the two plates. ***B***, Several milliliters of the uncured AD material (Shin-Etsu, KE-1300T) is applied to the bottom plate. ***C***, The top plate is then pressed down on top of the bottom plate. ***D***, Four hand clamps are used to secure the top plate to the bottom plate. To achieve a thinner AD thickness, the clamps are placed toward the middle. This creates a thickness of 100–200 μm in the center. ***E***, After 24 h, the cured AD material is in a thin sheet of the desired thickness. ***F***, A thin bead of silicone sealant (DOWSIL, 734 Flowable Sealant) is applied to the bottom surface of the insert before gently pressing it down onto the AD material. ***G***, Insert with AD attached. ***H***, Image of the circular insert with AD attached. The bulging section in the middle of the round insert rests on a lip inside the round chamber. In ***G*** and ***H***, the insert is oriented upside down with the AD material at the top.

The insert with the AD attached to the bottom fits tightly into the chamber and can be easily replaced, for example, in the event that the AD tears or breaks (Fig. 2*C*,*D*; Materials and Methods). The walls of the insert are 0.5–1 mm thick and can be made in various heights (e.g., 2.5–5 mm). This permits swapping out inserts to accommodate different distances to the cortical surface that may arise after surgical implantation of the chamber. The chamber and insert can be designed with a lip to restrict the maximum depth of the insert with respect to the bottom surface of the chamber (Figs. 2*A*, 3*H*).

The chamber is sealed between recordings by a 0.5-mm-thick silicone gasket (10:1 mixture of silicone to activator; Shin-Etsu, KE-1300T) which is placed on top of the chamber followed by a titanium cap that is screwed down to provide an airtight seal (Fig. 2*E–I*). This gasket is removed every time the chamber is opened for cleaning or recording and replaced with another sterile gasket, cap, and screws. The chamber is cleaned every 5 d or less by rinsing with sterile saline. The chamber is also rinsed with sterile saline before and after recording. Using this approach, we have not observed any evidence of infection in the four marmosets that have been implanted with this system.

The AD recording system is based on a previous design used in macaque recordings (Ruiz et al., 2013; Nassi et al., 2015; Nandy et al., 2017; Franken and Reynolds, 2021). A 10:1 mixture of silicone to activator (Shin-Etsu, KE-1300T) is placed in a mold, fixed with clamps, and left to cure for 24 h (Fig. 3*A–E*). The bottom surface of the insert (Fig. 2*C*) is slightly roughened using a file and then soaked in acetone to remove any debris or oils prior to attaching the AD material. A thin bead of silicone sealant (DOWSIL, 734 Flowable Sealant) is applied to the bottom surface of the insert. The insert is then pressed gently onto the AD which has been slightly stretched and placed on a flat surface (Fig. 3*F*). After 24 h, a scalpel is used to cut around the perimeter of the insert. Any excess AD that protrudes over the edge of the insert is trimmed (Fig. 3*G*,*H*). After autoclaving the insert is ready for use.

Recording chambers were implanted over area MT in two marmosets (both circular recording chambers) and over PFC in another two marmosets (one egg-shaped chamber and one circular chamber). Each animal was implanted with a headpost to stabilize the head for neurophysiological recordings and eye tracking. All surgical procedures were performed with the animal under general anesthesia in an aseptic environment in accordance with the recommendations in the Guide for the Care and Use of Laboratory Animals of the National Institutes of Health. All experimental methods were approved by the Institutional Animal Care and Use Committee (IACUC) of the Salk Institute for Biological Studies and conformed with NIH guidelines. Laminar probe recordings using 32-channel linear silicone electrode arrays (Atlas Neuroengineering) were performed in both marmosets with chambers over MT and one of the marmosets with a chamber over PFC. Neuropixels recordings were performed in both the marmosets with chambers over PFC.

### Single and multi-Neuropixels acute recording methods

**Figure 4.**
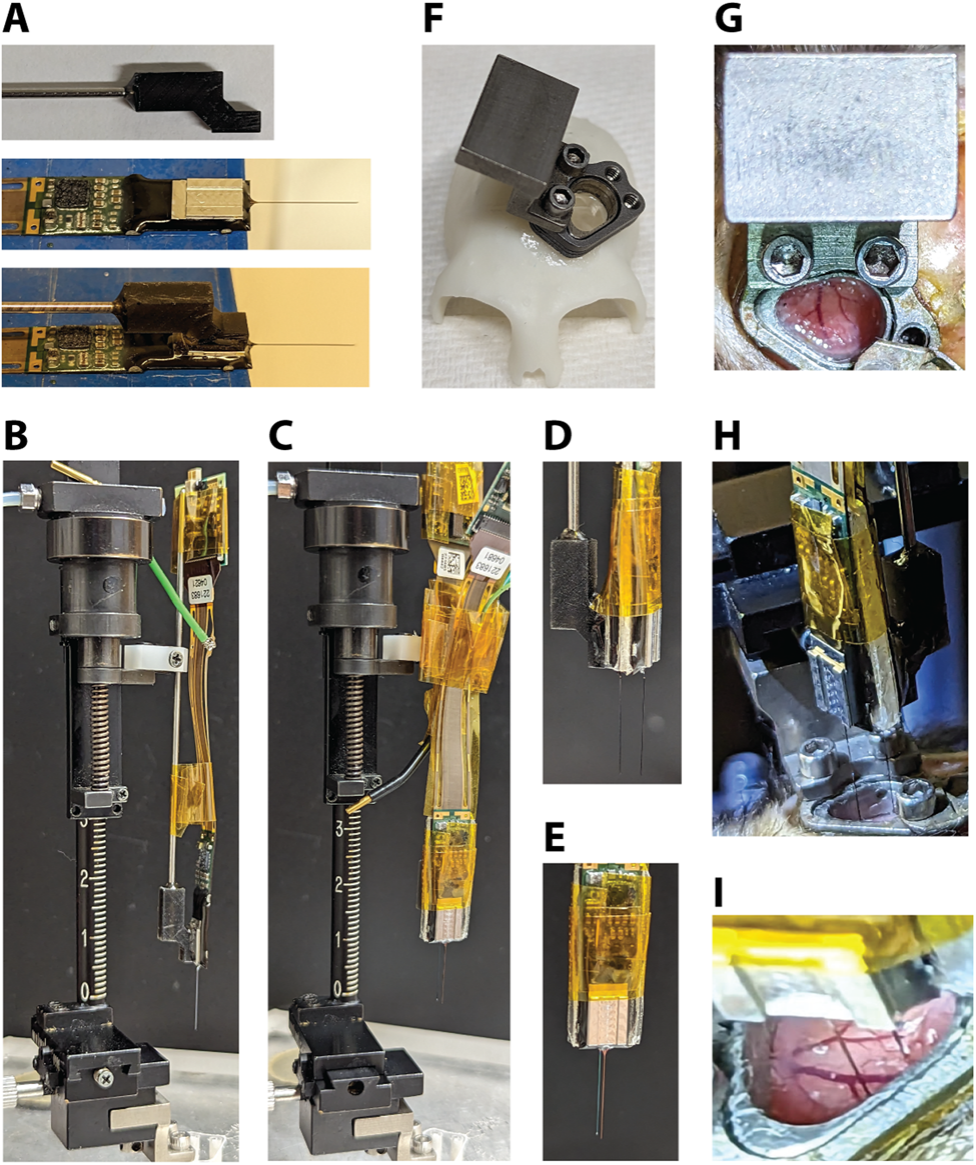
Neuropixels recording setup. ***A***, A custom 3D-printed single probe holder with a metal rod is glued to the base of the Neuropixels probe. Ground and reference wires are also attached. ***B***, The rod is held by the clamp of the microdrive. Tape is used to control the cable and headstage. ***C***, Example of double probe setup attached to microdrive (1.0 mm spacer). ***D***, Side view and (***E***) front view of double probe setup. ***F***, 3D-printed skull model made from a CT scan with a chamber and insert (AD attached) and a microdrive mount attached. ***G***, Photo of the microdrive mount when it is attached to the chamber. ***H***, Photo of the double probe setup during the process of lowering the probes (1.9 mm spacer). ***I***, Close-up view of the two probes while inserted in the cortex. Note the visible blood vessels that could be avoided during probe insertion.

Prior to recording, a custom 3D-printed single probe holder is glued (Gorilla glue, 2 part epoxy) to the base plate of the Neuropixels probe (Fig. 4*A*). A rod (1.13 mm diameter) is inserted into the single probe holder and glued in place (Gorilla glue, 2 part epoxy). Using standard parts, the single probe holder can then be mounted to a microdrive (Narishige, MO-97A oil hydraulic micromanipulator; Fig. 4*B*). To record from two locations within the chamber simultaneously, two Neuropixels probes are adhered to each other. Double-sided tape (Scotch, Double-Coated Tape) is placed on either side of a thin piece of plastic which serves as a spacer. To achieve the desired distance between the probes, spacers are made with different widths (used 1 mm or 1.9 mm). The spacer is sandwiched between the tail ends of the two probes, one of which has a single probe holder attached (Fig. 4*C–E*). Polyimide tape is then used to ensure the probes stay together and to keep the connector wires together.

Before attaching the microdrive, the outside of the chamber and the cap are thoroughly disinfected, and the cap is then removed using sterile tools. A microdrive mount made from titanium is then attached to the side of the chamber using the same screw holes as the cap (Fig. 4*F*,*G*). The microdrive holding the probe/s is then attached to the microdrive mount (Fig. 4*H*, also see Fig. 4*B*,*C*). Once the probes have been positioned over the desired recording location using the *x*–*y* stage of the microdrive, the probes are then slowly lowered into the brain through the AD (Fig. 4*H*,*I*; for more details about probe insertion, see Materials and Methods).

**Figure 5.**
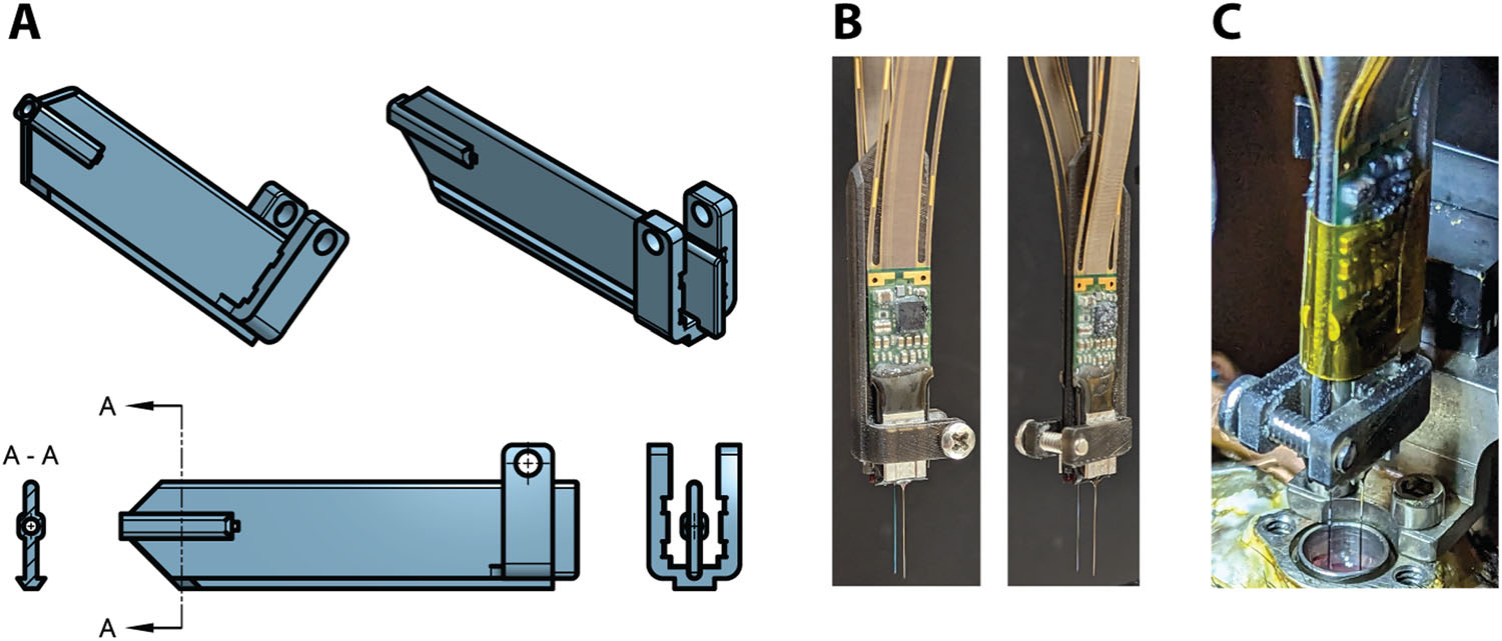
3D-printed two-probe holder. ***A***, 3D design drawings of the two-probe holder. The top row shows isometric views with the hole for the holding rod at the top and the probe clasps at the bottom. The bottom row shows a cutout view of the hole the holding rod is placed inside, a side view of the two-probe holder, and a view of the probe holder clasps which are shaped for the Neuropixels probes to slide into. ***B***, Example of the two-probe holder with two Neuropixels mounted. ***C***, Example of recording session using two-probe holder. Polyamide tape is added to control the cables and to add a secondary attachment to the holder. Probes are being lowered into a round chamber implanted in the PFC (right hemisphere).

After verifying that two probes could be easily inserted simultaneously, we developed a 3D-printed two-probe holder (Fig. 5*A*). The same size rod (1.13 mm diameter) used for the single probe holder design is inserted into a hole in the top of the 3D-printed two-probe holder and glued in place (Gorilla glue, 2 part epoxy). A Neuropixels probe is positioned on each side of the two-probe holder and locked in place using a screw (Fig. 5*B*). The spacing between probes can be determined by the thickness of the spacer (1 mm in this design). The probe is mounted and lowered in the same manner as the single probe holder design (Fig. 5*C*).

**Figure 6.**
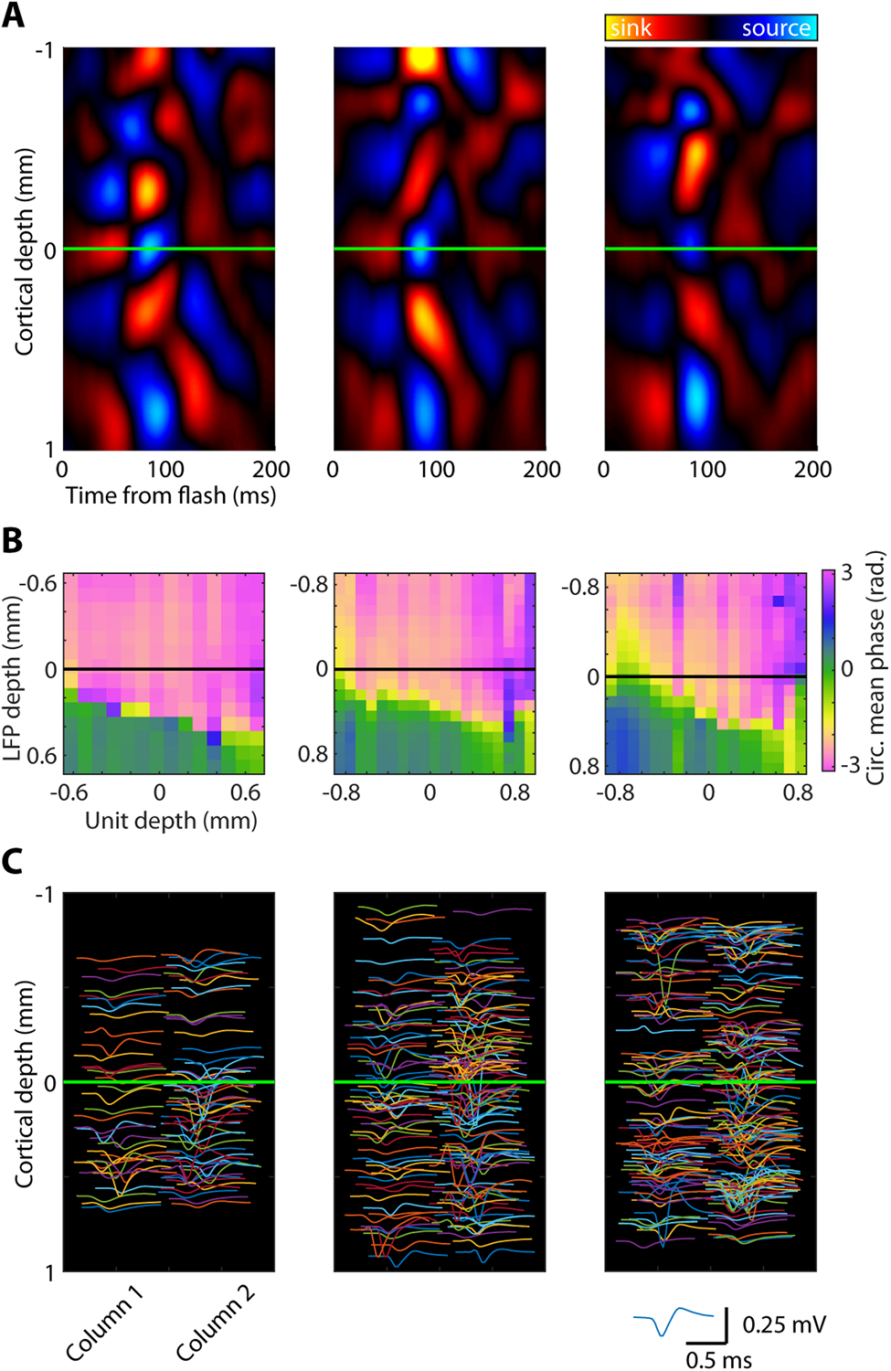
CSD and spike–phase relationships can be used to identify the input layer of the cortex. Each column shows an example of the CSD plot, spike–phase plot, and spiking data from an individual recording session using Neuropixels probes. ***A***, CSD plots (arb. units) with the middle of the input layer highlighted (green horizontal line). ***B***, Spike–phase plots with unit depths (spike) on the *x*-axis and LFP depths on the *y*-axis. Each entry shows the average spike–phase angle. The black line indicates the estimate of the middle of the input layer based on the corresponding CSD. The phase flip lines up approximately with the bottom of the input layer at the transition between layers IV and V. ***C***, Average waveforms for each detected unit are shown at the relative depth and relative location (column 1 or column 2) on the probe (jittered for visibility). The green horizontal line indicates the middle of the input layer determined using the CSD. Number of isolated units: left (*n* = 215), middle (*n* = 255), and right (*n* = 214).

After lowering the probe and allowing it to settle for ∼30 min, recordings are then conducted for ∼2 h while the marmoset performs a variety of tasks, including viewing and saccading to a perceptual illusion (Dotson et al., 2023), visual response field mapping, and full-screen flash sessions used to calculate the CSD. CSD plots are used to estimate laminar compartments based on the patterns of current sinks and sources and are calculated by taking the second spatial derivative of the average LFP response to a full-screen flash (Mitzdorf and Singer, 1978; Mitzdorf, 1985; Franken and Reynolds, 2021; Davis et al., 2023). We found that there was a common pattern of sources and sinks, with the earliest sink occurring near the middle of the cortex indicative of the “input” layer (layer 4) of the cortical column (Fig. 6*A*). We recently discovered a stereotyped spike–phase angle reversal at the boundary of the input and deep layers (Davis et al., 2023), which we used to verify our input layer estimates from the CSD plots. We see that the input layer as identified by the CSD corresponds to a position just above the phase angle reversal (Fig. 6*B*). Unit activity was reliably found to occur within a millimeter above and below the estimated input layer (Fig. 6*C*).

**Figure 7.**
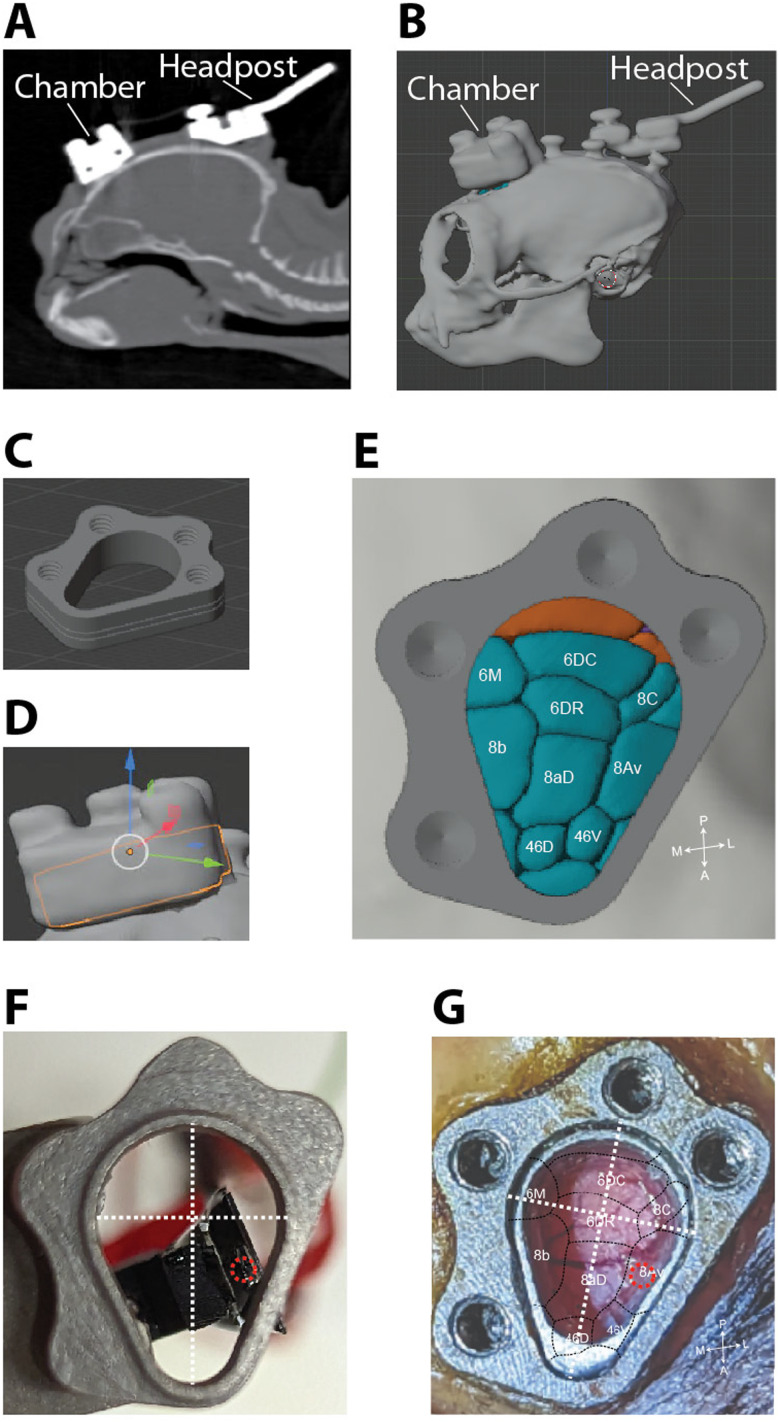
Reconstruction of recording locations. Recording locations were identified by combining 3D models and images taken of the probe and chamber. ***A***, CT scan after head post and chamber implant. ***B***, 3D model of skull created from CT scan. ***C***, 3D model of chamber. ***D***, Chamber model aligned to the chamber on the 3D model of skull. The chamber model is transparent with an orange outline. ***E***, Image through chamber of 3D brain model (Liu et al., 2021; marmosetbrainmapping.org). Anterior (A), posterior (P), medial (M), and lateral (L) directions are indicated in the bottom right corner. ***F***, ***G***, A separate chamber is used as a reference to determine the probe location relative to the chamber. After each recording, the microdrive is mounted on a pedestal and chamber identical to the ones used for recording (***F***). A picture is taken from below and then annotated. The chamber references (white lines) and probe shank location (red circle) are placed on top of the reconstructed map of areas to identify the recording location (***G***). This particular recording session is likely in area 8aV. Double probe recording sessions are reconstructed in the same manner.

To reconstruct the location of the recording chamber with respect to the cortex, we first built a 3D model of the skull made from a CT scan taken after the headpost and chamber were implanted (Fig. 7*A*,*B*) and then aligned a 3D model of the chamber (Fig. 7*C*,*D*). We then registered a separate 3D model of the skull (made from a CT scan taken before implanting), with a 3D model of the marmoset brain inside (Liu et al., 2021; marmosetbrainmapping.org), to the implanted skull model. After digitally removing the skull, we could estimate the location of each cortical area with respect to the chamber (Fig. 7*E*). To estimate the location of the probe after each recording session, the microdrive is mounted on a pedestal and chamber identical to the ones used for recordings, and a picture is taken from below (Fig. 7*F*). This provides the location of the probe with respect to the chamber. This information can be used to identify the location and cortical area the probe likely penetrated (Fig. 7*G*).

## Discussion

The shape of the marmoset cortex provides a unique opportunity to study laminar processing. Here we demonstrate an efficient method for acute Neuropixels recordings in the behaving marmoset. The AD recording system enables repeatable acute recordings with high optical visibility to avoid damaging blood vessels and a low risk of infection over an extended period of time. This technique has been successfully combined with marmosets performing a variety of complex behavioral tasks (Dotson et al., 2023), to enable an exceptional ability to study laminar processing in a primate. The recording system is also highly versatile. Using the same hardware, single microelectrodes can be used to microstimulate. This is particularly useful for identifying cortical areas such as the frontal eye fields.

While the method presented here offers numerous benefits, there are also limitations including having to perform routine removal of granulation tissue (see Materials and Methods for more details). In some instances, it was not feasible to place the AD directly on the pia, leaving a small fluid-filled gap. This gap did not prove to be a problem for probe insertion but it may have shortened the window for recording by allowing tissue growth to more easily occur on the pial surface. Although the chambers used here were flat on the bottom, using form-fitting chambers (Dotson et al., 2015, 2017; Jendritza et al., 2023) and inserts may help to achieve a smaller gap and thus extend recording periods.

With some modifications, this method could be integrated with optical imaging (Pattadkal et al., 2022; Song et al., 2022) and optogenetics (Macdougall et al., 2016; Komatsu et al., 2017; Ebina et al., 2019; Jendritza et al., 2023; Obara et al., 2023) in the head-fixed marmoset. Another avenue for future work will be to combine this technique for acute Neuropixels recordings with semichronic (Dotson et al., 2015, 2017, 2018; Pomberger and Hage, 2019; Jendritza et al., 2023) and wireless recording methods (Courellis et al., 2019; Dotson and Yartsev, 2021; Mao et al., 2021; Walker et al., 2021; Piza et al., 2023; Wong et al., 2023) to understand the laminar mechanisms of visual motion and self-motion perception during natural movements in three dimensions. Chambers and semichronic microdrives capable of housing Neuropixels probes may be designed using CT scans to fit specific monkeys and target-specific cortical regions (Dotson et al., 2015, 2017; Jendritza et al., 2023).

## Synthesis

Reviewing Editor: Christine Portfors, Washington State University Decisions are customarily a result of the Reviewing Editor and the peer reviewers coming together and discussing their recommendations until a consensus is reached. When revisions are invited, a fact-based synthesis statement explaining their decision and outlining what is needed to prepare a revision will be listed below. The following reviewer(s) agreed to reveal their identity: Maureen Hagan.

Overall, the chamber design was described in great details. More details in the maintenance of the system and probe insertion procedures would help replicating this technique in other labs.

Major:

1. About maintenance of the AD system

a. How often did the insert with AD need to be replaced (e.g. in the event that the AD tears or breaks)?

b. It would be great to include more details on tissue growth underneath AD.

i. Did it happen in all four monkeys?

ii. How often did the authors remove the tissue?

iii. In the abstract, "This technique enables repeated acute penetrations over a period of several months. With occasional removal of tissue growth on the pial surface, recordings can be performed for a year or more." Does it mean that the authors were able to record without any tissue regrowth in the first several months?

c. What is the cleaning procedure when replacing the AD? The methods state that every 5 days a most, the cap and gasket are removed, the chamber rinsed with sterile saline, and then a sterile gasket and chamber are replaced. But when the AD is removed, is there a procedure you use for cleaning the pial surface before placing a new AD?

d. On a related note, you mention reduced risk of infection but not responses to infections. How often did you get infections below the AD? Were you able to successfully control them?

e. Infections above the AD are presumably of less welfare concern, but did you have any problems with bacteria growth above the AD?

2. About probe insertion procedures

a. What was the typical insertion speed?

b. How were probes sterilized/cleaned prior to insertion?

c. Was there any dimpling when popping through the AD?

d. I think it's important to document the explanting procedure as well, e.g. How fast did the authors explant the probe? Did the authors clean the chamber after explanted? How did the authors clean and store the probes? If the authors followed some standard procedures, it would be good to include the reference.

Minor:

1. Will the CAD files of the chamber be available online?

2. Were the chambers 3D printed or machined?

3. What kind of laminar probe was used in MT?

4. How did the authors make the silicone gasket?

5. Did the authors put sterile screws on every time they open the chamber? It mentioned sterile gasket and cap were used in the text but it's not clear about the screws.

6. Page 4: "short, linear and sharpened NHP version" first use of NHP

7. What is the weight of the chamber/gasket/cap system?
